# Aquaporin Expression and Water Transport Pathways inside Leaves Are Affected by Nitrogen Supply through Transpiration in Rice Plants

**DOI:** 10.3390/ijms19010256

**Published:** 2018-01-16

**Authors:** Lei Ding, Yingrui Li, Limin Gao, Zhifeng Lu, Min Wang, Ning Ling, Qirong Shen, Shiwei Guo

**Affiliations:** 1 Jiangsu Provincial Key Lab for Organic Solid Waste Utilization, National Engineering Research Center for Organic-Based Fertilizers, Jiangsu Collaborative Innovation Center for Solid Organic Waste Resource Utilization, Nanjing Agricultural University, Nanjing 210095, China; lei.ding@uclouvain.be (L.D.); yrli@psc.ac.cn (Y.L.); limingao@njau.edu.cn (L.G.); luzhifeng@njau.edu.cn (Z.L.); minwang@njau.edu.cn (M.W.); nling@njau.edu.cn (N.L.); shenqirong@njau.edu.cn (Q.S.); 2 Institute des Sciences de la Vie, Université catholique de Louvain, B-1348 Louvain-la-Neuve, Belgium

**Keywords:** photosynthesis, nitrogen, aquaporins, transpiration, leaf hydraulic conductance

## Abstract

The photosynthetic rate increases under high-N supply, resulting in a large CO_2_ transport conductance in mesophyll cells. It is less known that water movement is affected by nitrogen supply in leaves. This study investigated whether the expression of aquaporin and water transport were affected by low-N (0.7 mM) and high-N (7 mM) concentrations in the hydroponic culture of four rice varieties: (1) Shanyou 63 (SY63), a hybrid variant of the *indica* species; (2) Yangdao 6 (YD6), a variant of *indica* species; (3) Zhendao 11 (ZD11), a hybrid variant of *japonica* species; and (4) Jiuyou 418 (JY418), another hybrid of the *japonica* species. Both the photosynthetic and transpiration rate were increased by the high-N supply in the four varieties. The expressions of aquaporins, plasma membrane intrinsic proteins (*PIPs*), and tonoplast membrane intrinsic protein (*TIP*) were higher in high-N than low-N leaves, except in SY63. Leaf hydraulic conductance (K_leaf_) was lower in high-N than low-N leaves in SY63, while K_leaf_ increased under high-N supply in the YD6 variant. Negative correlations were observed between the expression of aquaporin and the transpiration rate in different varieties. Moreover, there was a significant negative correlation between transpiration rate and intercellular air space. In conclusion, the change in expression of aquaporins could affect K_leaf_ and transpiration. A feedback effect of transpiration would regulate aquaporin expression. The present results imply a coordination of gas exchange with leaf hydraulic conductance.

## 1. Introduction

Nitrogen (N) is an essential macro-nutrient for plants. It affects many aspects of plant growth and development, including water uptake and photosynthesis [1]. It was shown that the photosynthetic rate and CO_2_ transport conductance increased under high-N supply [2,3]. Meanwhile, water use efficiency was 22% and 26% higher under high-N than low-N supply in SY63 and YD6 rice cultivars, respectively. Interestingly, it was shown that leaf water potential decreased under high-N treatment in comparison with low-N treatment in SY63, but not in YD6 [4]. Aquaporin expression was not increased by high-N supply in SY63 [3,4]. In roots, a high-N supply increased water uptake ability and enhanced the expression of aquaporin and root hydraulic conductance [4,5].

In the soil–plant–atmosphere continuum system, water moves from the soil to the root xylem through the apoplast (i.e., cell wall space) and/or the cell-to-cell pathway [6,7]. After moving inside xylem vessels, water is delivered to the whole leaf lamina before evaporating through the stomata complex. Water movement inside leaves or leaf hydraulic conductance (K_leaf_) includes two aspects: (1) water movement through the leaf xylem (i.e., petiole and venation) and (2) water movement outside the xylem (i.e., bundle sheath and mesophyll) [8,9]. Once water exits the leaf xylem, it enters the bundle sheath. This is then followed by movement through apoplastic, symplastic and gas phase pathways in the mesophyll [9]. However, the dominant pathway probably differs among species and conditions, i.e., temperature, irradiance and drought stress [8,10,11].

K_leaf_ was highly variable, ranging from 0.76 to 49 mmol·m^−2^·s^−1^·MPa^−1^ in 107 tested species [8]. The large variablility of K_leaf_ was partially or mainly contributed to by variations in leaf anatomy development [9,12,13]. In the *Oryza* genus, Xiong et al. [14] observed a positive correlation between the conductance outside xylem (K_ox_) and the following factors: leaf vein length, intercellular air space (*f*_IAS_), mesophyll cell surface area exposed to intercellular air space (Sm), and chloroplast exposed to intercellular air space (Sc). Meanwhile, they found that K_ox_ was negatively correlated to cell wall thickness.

Leaf anatomy was more associated with water movement inside the xylem and/or the apoplastic and gas phase pathways. Nonetheless, the symplastic pathway could limit water moving from cell to cell because the process requires water to pass through a high resistance cell membrane or apoplastic barriers. However, the presence of aquaporins in the membrane could reduce the resistance and facilitate water transport [11,15,16]. It was demonstrated that K_leaf_ decreased after silencing the aquaporin expression in bundle sheath cells [17]. Aquaporin also played important roles in regulating K_leaf_ under drought stress [8,10].

Transpiration pull drives the water movement within the whole plant. Therefore it is reasonable to suggest that a potential correlation exists between transpiration and aquaporin function in both the roots and shoots. This was reviewed in “Plant Aquaporin and Transpiration” by Maurel et al. [18]. Changes in tissue hydraulics mediated by root and shoot aquaporins can indirectly impact plant transpiration. Alternatively, it was pointed out that a feedback effect of transpiration on aquaporin function exists. Both positive and negative effects were demonstrated regarding how transpiration regulates aquaporin function [10,19,20,21].

Little data shows how leaf water status is affected by N supply. Similarly, little is known about the coordination between water movement and photosynthetic CO_2_ fixation regulated by N. In this study, we focused on how nitrogen supply affected the expression of aquaporin in leaves, indicating a contribution by the symplastic water pathway. The correlation between transpiration and aquaporin function was discussed.

## 2. Results

### 2.1. Gas-Exchange Was Affected by N Supply

**Figure 1:**
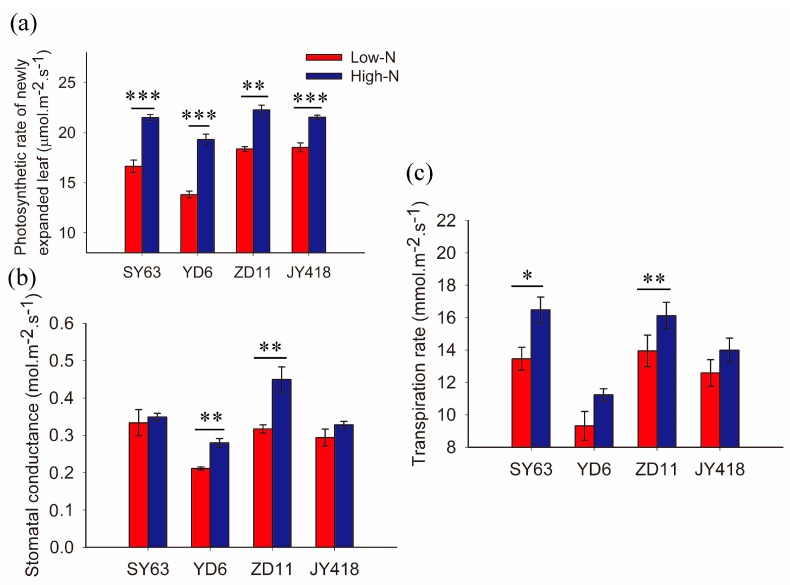
Effects of different nitrogen supply levels on (**a**) photosynthetic rate, (**b**) stomatal conductance and (**c**) transpiration rate in four rice cultivars, namely Shanyou 63 (SY63), Yangdao 6 (YD6), Zhendao 11 (ZD11) and Jiuyou 418 (JY418). Two nitrogen concentrations were supplied as low-N (0.7 mM) and high-N (7 mM) treatments in hydroponic culture. In each treatment and genotype, four plants were measured with newly expanded leaves. The data represents the means ± SE. Significant differences between treatments are indicated by *, ** and ***, at levels of *p* < 0.05, *p* < 0.01 and *p* < 0.001, respectively.

In this study, low- and high-N treatments were applied in four rice varieties. The light-saturated photosynthetic rate was measured with newly expanded leaves. Compared with low-N treatment, high-N supply significantly increased the photosynthetic rate. This was enhanced by 30%, 40%, 15% and 16% in SY63, YD6, ZD11 and JY418, respectively (Figure 1a). In both low- and high-N treatments, the photosynthetic rate was lowest in YD6 compared to the other varieties.

In YD6 and ZD11, stomatal conductance (g_s_) was increased by high-N supply (Figure 1b). In SY63 and JY418, no difference of g_s_ was observed between low-N and high-N treatments. The transpiration rate (Tr) was higher under high-N treatment than low-N in SY63 and ZD11. No significant difference of Tr was found between high-N and low-N treatments in YD6 and JY418 (Figure 1c).

### 2.2. Aquaporin Expression Was Affected by N Supply

**Figure 2:**
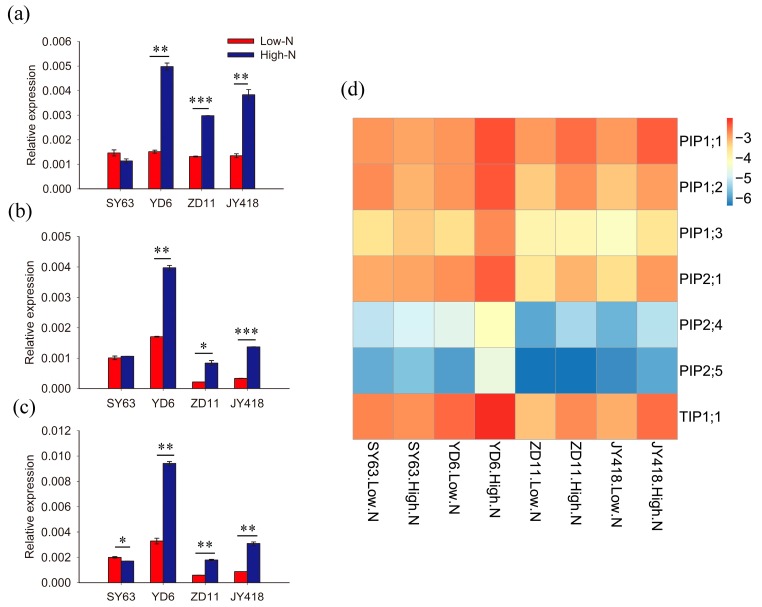
Effects of different nitrogen supply levels on the expression of (**a**) *OsPIP1;1* (**b**) *OsPIP2;1* and (**c**) *OsTIP1;1* in four rice cultivars. Two nitrogen concentrations were supplied as Low-N (0.7 mM) and High-N (7 mM) in hydroponic culture. Leaves were harvested from three plants and then total RNA was extracted for RT-qPCR. Three independent reactions were prepared for each sample. The data represents the means ± SE. Significant differences between treatments are indicated by *, ** and ***, at levels of *p* < 0.05, *p* < 0.01 and *p* < 0.001, respectively.

To examine the effect of N supply on aquaporin expression in leaves, the expression of six PIP and one TIP gene (*OsPIP1;1*, *OsPIP1;2*, *OsPIP1;3*, *OsPIP2;1*, *OsPIP2;4*, *OsPIP2;5* and *OsTIP1;1*) were detected by real-time-quantitative PCR (RT-q-PCR) according to Sakurai-Ishikawa et al. [22,23]. It was demonstrated that *OsPIP2;1* and *OsPIP2;5* could facilitate water transport when expressed in yeast. *OsPIP1;1*, *OsPIP1;2*, *OsPIP1;3* and *OsPIP2;1* were mainly localized in mesophyll cells [22]. The expression of *OsPIP2;4* and *OsPIP2;5* was lower when compared to the other genes (Figure 2d). The expression of all genes was higher in high-N than low-N treatment in YD6, ZD11 and JY418 leaves (Figure 2). In SY63, no higher expression of *OsPIP1;1*, *OsPIP2;1* and *OsTIP1;1* was observed in high-N than in low-N treatment. Instead, *OsTIP1;1* expression was significantly lower in high-N than low-N treatment (Figure 2c).

### 2.3. Hydraulic Conductance Was Affected by N Supply

**Figure 3:**
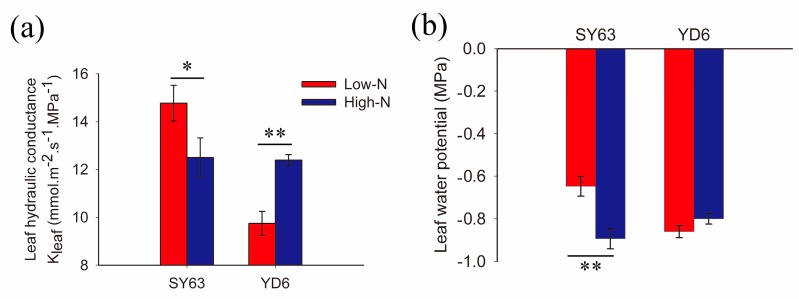
The comparison of (**a**) leaf hydraulic conductance and (**b**) leaf water potential, between low-N and high-N supply in SY63 and YD6 cultivars. Four plants were measured in each treatment and genotype. The data represents the means ± SE. Significant differences between treatments are indicated by * and **, at levels of *p* < 0.05 and *p* < 0.01, respectively.

There was coordination between K_leaf_ and photosynthesis [8]. To determine the effect of nitrogen supply on water movement inside leaves, K_leaf_ was compared between high-N and low-N treatments in SY63 and YD6. In SY63, K_leaf_ decreased under high-N compared to low-N treatment. Meanwhile an increase of K_leaf_ was observed in YD6 under high-N treatment (Figure 3a). Leaf water potential was more negative in SY63 under high-N than low-N treatment. There was no difference of leaf water potential between high-N and low-N treatment in YD6 (Figure 3b).

### 2.4. The Relationship between Aquaporin Expression, Transpiration and Cell Wall Thickness

**Figure 4:**
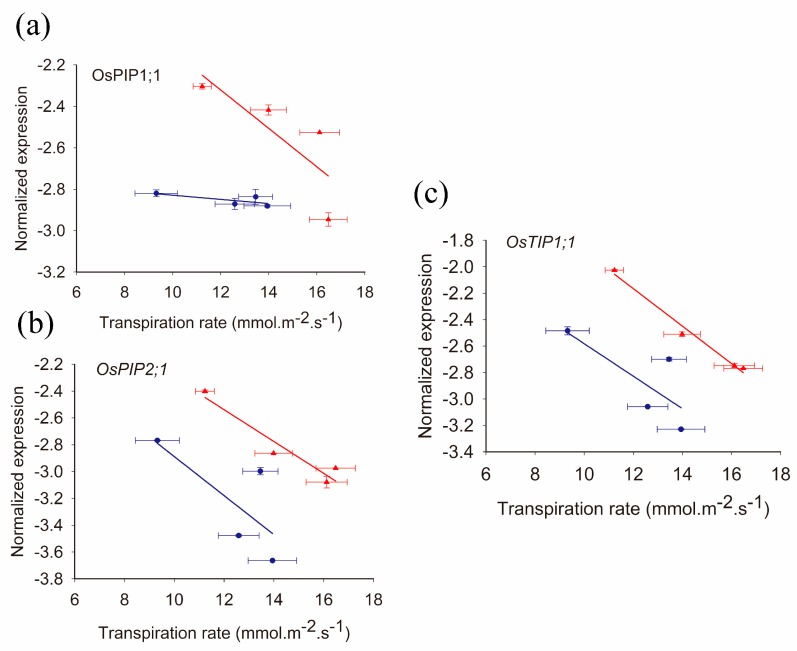
The correlation between transpiration rate and the expression of (**a**) *OsPIP1;1* (**b**) *OsPIP2;1* and (**c**) *OsTIP1;1* under low-N (blue circle) and high-N (red triangle). The data represents the means ± SE. The correlations were listed as follows: (**a**) low-N treatment: *y* = −0.01*x* − 2.7275, *R*^2^ = 0.53; high-N treatment: *y* = −0.09*x* − 1.2083, *R*^2^ = 0.64. (**b**) low-N treatment: *y* = −0.15*x* − 1.4333, *R*^2^ = 0.53; high-N treatment: *y* = −0.12*x* − 1.1143, *R*^2^ = 0.92, *p* < 0.05. (**c**) low-N treatment: *y* = −0.12*x* − 1.3526, *R*^2^ = 0.57; high-N treatment: *y* = −0.14*x* − 0.4634, *R*^2^ = 0.98, *p* < 0.01.

To know the relationship between aquaporin expression and transpiration among rice varieties, three genes (i.e., *OsPIP1;1*, *OsPIP2;1* and *OsTIP1;1*) were investigated. Under high-N supply, a significant negative correlation was observed between transpiration rate and the expression of both *OsPIP2;1* and *OsTIP1;1*, particularly *OsTIP1;1* in four varieties (*R*^2^ = 0.98). Under low-N supply, the correlation was weaker in comparison with high-N treatment (Figure 4).

**Figure 5:**
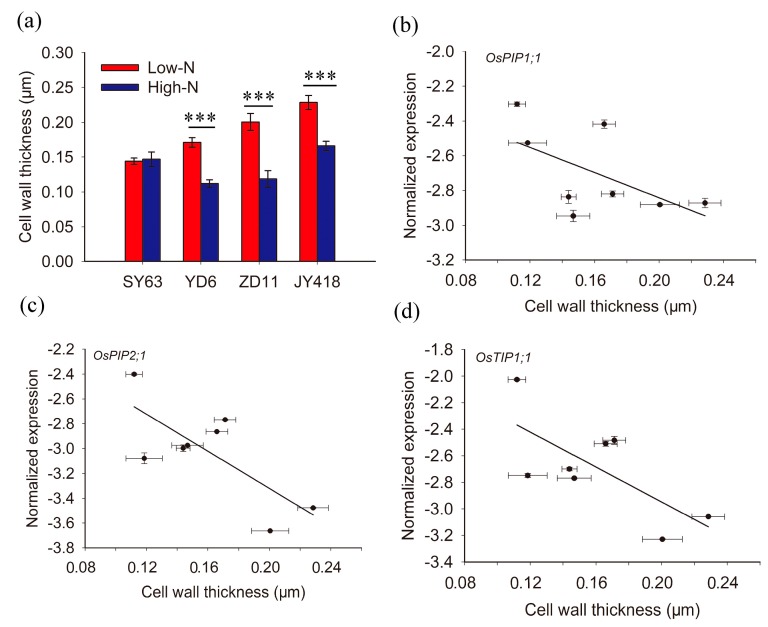
(**a**) The effect of nitrogen supply on cell wall thickness and the correlation between cell wall thickness and the expression of (**b**) *OsPIP1;1* (**c**) *OsPIP2;1* and (**d**) *OsTIP1;1*. Cell wall thickness was measured (*n* > 20) by ImageJ software (National Institutes of Health, Bethesda, MD, USA http://imagej.nih.gov/ij) with images from a transmission electron microscope. The data represents the means ± SE, Significant differences between treatments are indicated by ***, at a level of *p* < 0.001. The correlations were listed as follows: (**b**) *y* = −3.62*x* − 2.118, *R*^2^ = 0.34; (**c**) *y* = −7.48*x* − 1.825, *R*^2^ = 0.56, *p* < 0.05; (**d**) *y* = −6.58*x* − 1.632, *R*^2^ = 0.50.

It was shown that cell wall thickness was negatively correlated with K_ox_ [14]. To better understand how nitrogen affects water movement inside leaves, cell wall thickness was measured and a correlation between cell wall thickness and aquaporin expression was demonstrated. In SY63, no difference in cell wall thickness was observed between high-N and low-N supplied plants. However, cell wall thickness decreased significantly under high-N treatment in YD6, ZD11 and JY418 (Figure 5a). A significant negative correlation was observed between cell wall thickness and the expression of *OsPIP2;1* (Figure 5c) (*R*^2^ = 0.56, *p* < 0.05). Meanwhile the correlation was not significant between cell wall thickness and the expression of *OsPIP1;1* (Figure 5b) and *OsTIP1;1* (Figure 5d).

### 2.5. The Relationship between Transpiration Rate and Intercellular Air Space (f_IAS_)

**Figure 6:**
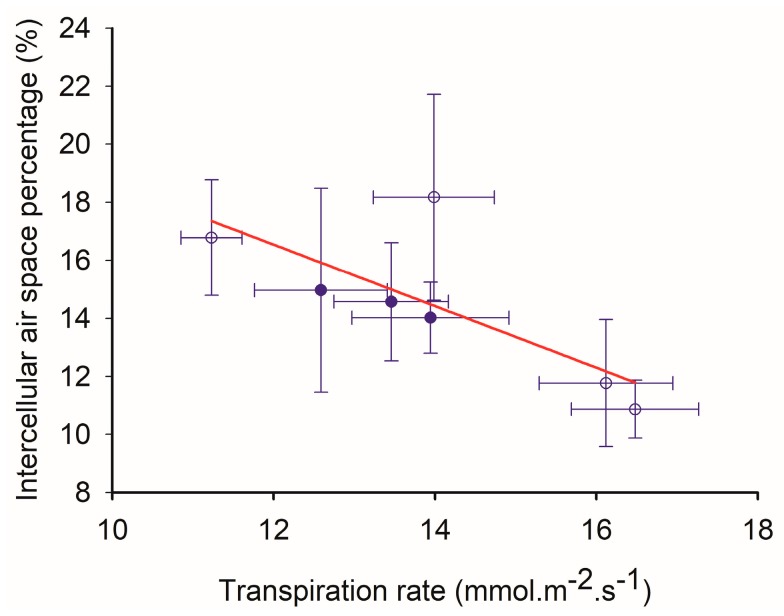
The correlation between transpiration rate and intercellular air space. The correlation was listed as follows: *y* = −1.06*x* + 29.26, *R*^2^ = 0.58, *p* < 0.05. Filled and opened circles indicated low-N and high-N treatment, respectively.

Once water exits the leaf xylem, it enters the bundle sheath. It then moves through apoplastic, symplastic and gas phase pathways in mesophyll. Aquaporins play an important role in the symplastic pathway water flow. Meanwhile, *f*_IAS_ affects water moving in the gas phase and apoplastic pathways. To further understand the correlation between transpiration and leaf anatomy, *f*_IAS_ was analyzed. A significant negative correlation was observed between transpiration rate and *f*_IAS_ (*R*^2^ = 0.58) (Figure 6).

## 3. Discussion

### 3.1. N Supply Increase Photosynthetic CO_2_ Fixation

The present results demonstrated that the light saturated photosynthetic rate was increased by high-N supply (Figure 1a). N plays an important role in the process of photosynthetic CO_2_ fixation [24,25], and there was a positive correlation between photosynthetic rate and nitrogen content in leaves [24]. Approximately 80% of total leaf N is located inside chloroplasts [26], where CO_2_ is fixed. Nonetheless, it was shown that N content inside photosynthetic apparatus was usually extra enough to fix CO_2_ under low- and high-N supply. The high photosynthetic rate was attributed to increased CO_2_ transport conductance and the high chloroplastic CO_2_ concentration under high N [2]. More N was present in its inactivated form inside the chloroplast, especially under high N supply. Indeed, it was demonstrated that photosynthesis is limited by CO_2_ availability inside the chloroplast. Chloroplastic CO_2_ availability is determined by g_s_ and mesophyll conductance (g_m_) under light saturated conditions [3,27,28].

### 3.2. N Supply Affecting Aquaporin Expression and Hydraulic Conductance

High-N supply increased the expression of PIPs and TIP in YD6, ZD11 and JY418 (Figure 2), indicating that water movement inside the leaves (i.e., K_leaf_) was affected by N supply. K_leaf_ was higher in high-N than low-N plants in the YD6 variant. In comparison, K_leaf_ decreased under high-N supply in SY63 (Figure 3a). It was shown that there was higher root porosity and root aerenchyma in the SY63 plant supply with high-N in comparison with the YD6 plant [4]. The high aerenchyma formation would restrict water transport in roots [29], which further affects aerial tissue water supply and K_leaf_. Leaf water potential decreased in SY63 plants supplied with high-N when compared with low-N supply plants, while it was not observed in YD6 plants (Figure 3b). Additionally, there was no increase in expression of both PIPs and TIP under high-N supply in the SY63 cultivar, which probably further resulted in K_leaf_ decrease.

To understand the water movement inside leaves (K_leaf_), two pathways exist: (1) water movement through the leaf xylem (i.e., petiole and venation) and (2) water movement outside the xylem (i.e., bundle sheath and mesophyll) [8]. The resistance of the former pathway is small and K_leaf_ was more restricted by later conductance. Once water exits the leaf xylem, it enters the bundle sheath. It then goes through apoplastic, symplastic and gas phase pathways [9]. Aquaporins play important roles in both the processes of water entering from xylem vessels to the bundle sheath and of water’s transcellular movement outside bundle sheath [9,17]. It was demonstrated that K_leaf_ decreased after silencing the expression of aquaporin in bundle sheath cells. Under high-N supply, the increase of aquaporins expression could facilitate more water transport through the membrane of both bundle sheath and mesophyll cells. Additionally, it was shown that cell wall thickness was negatively correlated with K_ox_ [14]. In this study, cell wall thickness decreased significantly under high-N supply compared with low-N supply in YD6, ZD11 and JY418 (Figure 5a). Indeed, there was a significantly negative correlation between cell wall thickness and the expression of *OsPIP2;1* (Figure 5c). The results indicated that the increase of K_leaf_ was partially explained by a lower cell wall thickness under high-N treatment than with low-N treatment. However, no difference of cell wall thickness was observed between high-N and low-N treatments in SY63, which might be due to the decrease of K_leaf_ under high-N treatment.

Aquaporins facilitate not only water transport, but also the transport of other small solutes [16,30], such as CO_2_ [27,31]. High-N supply increased the expression of aquaporin in leaves (Figure 2), and it was shown that CO_2_ transport conductance was higher in high-N supply than low-N supply cultivars [2,3]. It was proposed that more expressed aquaporin under high-N supply assisted CO_2_ in passing through the membrane, resulting in the increase of g_m_. However, there was no increase of aquaporin expression (Figure 2), but g_m_ increased under high-N supply in SY63 [2]. This indicates that g_m_ could be regulated by other factors. It was shown that high g_m_ was due to a greater chloroplast size (i.e., a shorter distance to the plasma membrane), which resulting resulted in the CO_2_ transport resistance in the liquid phase to be lower in high-N than low-N leaves.

Meanwhile, N could also affect the expression of aquaporin in roots. In this study, high-N enhanced the expression of *PIPs* (Figure S1). It was shown that N deprivation decreased the expression of aquaporins in rice roots, whereas N resupply increased the expression of aquaporins [5]. In other studies, similar results were obtained showing that high NO_3_^−^ supply could increase the expression of aquaporin in roots or root hydraulic conductivity (Lpr) [32,33,34]. Our result demonstrated that Lpr was increased by high-N supply (Figure S2). In the process of root cortex radial water movement, the contribution of aquaporin is generally high, going up to 79% in well-watered conditions and 85% under drought stress in rice plants [35]. The expression of *OsPIPs* was higher in high-N than low-N in roots (Figure S1). This could result in high Lpr. Additionally, the difference in Lpr was also correlated with the differences in root anatomy development in high-N and low-N treatments [4]. It was shown that more aerenchyma formation and lignification could restrict radial water movement in low-N supply roots.

### 3.3. The Interaction between Transpiration and Aquaporin Expression

A different increase in Tr was observed under high-N supply in the four varieties (Figure 1). Under high-N supply, the increase in Tr aimed to (1) cool down the plants as it was shown that there was low leaf temperature in plants supplied with high-N [36] and the low temperature correlated with high Tr [37]; (2) absorb more N; and (3) fix more carbon (C). In the soil–plant–atmosphere continuum, water goes uphill based on the root pressure and transpiration pull. In this process, nitrogen (N) was absorbed from soil and carbon was fixed from the atmosphere. Under high-N supply, more water was utilized for photosynthetic CO_2_ fixation.

Aquaporin could regulate transpiration in plants [18]. Under high-N supply, the increased expression of *PIPs* and *TIP* could contribute to the high Tr. It was demonstrated that genetic manipulation of aquaporins can dramatically decrease or enhance Tr or g_s_, by up to 30–40% [18]. Indeed, aquaporin could directly regulate movement of guard cells, the closure of stomata and further affect Tr. It was demonstrated that aquaporin facilitate H_2_O_2_ entering the guard cell, induce the closure of stomata under abscisic acid (ABA) and are involved in pathogen treatment [38]. In the other study, Grondin et al. [39] showed that aquaporin contributed to ABA-triggered stomatal closure through phosphorylation of *PIP2;1*. Additionally, down-regulation of aquaporins in the bundle sheath could also induce stomata closure and Tr decrease [40]. The contribution of aquaporins to transpiration control goes far beyond the issue of water transport during stomatal movements. It involves emerging cellular and long-distance signaling mechanisms which ultimately act on plant growth [18].

There was a feedback effect of transpiration on aquaporin function. The results of this study showed that a negative correlation was observed between the transpiration rate and the expression of aquaporins in the four rice varieties (Figure 4), indicating that transpiration traits could affect the expression of aquaporins inside leaves. Similar results were found by Morillon and Chrispeels [19] for the genus Arabidopsis. Transpiration was down-regulated by ABA and high humidity, and they found that the osmotic water permeability (Pos) of mesophyll protoplasts increased. This indicated that there was higher aquaporin activity. It was proposed that cell-to cell movement of water would be accelerated by an increase in Pos to support the high rate of xylem to phloem water recycling when there is no or little transpiration, while under high transpiration conditions, apoplastic flux mainly contributed to the water movement inside leaves. Again in maize plants, there were higher aquaporin protein levels in leaves and a lower transpiration rate or g_s_, which was affected by endogenous ABA levels inside leaves [20]. In some studies, positive feedback was observed and they found a positive relationship between aquaporin expression and Tr or g_s_, especially under drought stress [10,41]. In roots, more positive feedback was demonstrated. In rice plants, the *OsPIP2;5* gene was shown to be specifically responsive to transpiration because its mRNA and protein accumulate in roots during the day. This was reduced when shoots were exposed to high humidity (low transpiration) [23]. In the present studies, high aquaporin expression in roots could be caused by both the feedback effect from transpiration and local high N supply.

Additionally, a negative correlation was observed between the transpiration rate and *f*_IAS_ (Figure 6), which indicates that water movement resistance is increased by a small *f*_IAS_ in cultivars with a high transpiration trait. It was shown that there was a positive correlation between *f*_IAS_ and K_ox_ in plants of genus *Oryzas* [14]. Additionally, under most conditions, apoplastic and gas phase water movement provide the majority of conductance outside the bundle sheath [9]. Combined with the correlation between transpiration rate, aquaporin expression and *f*_IAS_, it was proposed that K_ox_ was low in cultivars with a high transpiration trait, to avoid more water loss.

There was an opposite correlation between the transpiration rate and aquaporin expression amongst genotypes and plants with different N treatments. In plants with high transpiration rates, it was proposed that plants attempt to increase water use efficiency through decreasing the expression of aquaporin and intercellular air space, as well as increasing cell wall thickness to further decrease K_leaf_. Under high-N supply, photosynthetic CO_2_ fixation was enhanced. Meanwhile more water demand (high transpiration rate) was required. This was achieved through increasing K_leaf_ and the expression of aquaporin. Yet, a more negative correlation was demonstrated between the transpiration rate and the expression of aquaporin under high-N compared with low-N treatment, indicating that plants attempt to increase water use efficiency in accordance with big water demands.

## 4. Materials and Methods

### 4.1. Plant Materials

Four rice cultivars were used in this study: (1) Shanyou 63 (SY63), a hybrid variant of the *indica* species; (2) Yangdao 6 (YD6), a variant of *indica* species; (3) Zhendao 11 (ZD11), a hybrid variant of *japonica* species; and (4) Jiuyou 418 (JY418), another hybrid of the *japonica* species. Our previous studies mainly focused on SY63 and YD6 to demonstrate the effect of nitrogen supply on photosynthetic efficiency and root water uptake [3,4,42]. ZD11 and JY418 were widely used in local place and ZD11 was used in our field experiment [43,44]. Therefore we selected these four variants in the present study. Two nitrogen level treatments were applied with equal ratio of ammonium and nitrate, including 0.7 mM (Low-N) and 7 mM (High-N). Rice seeds were disinfected in 10% H_2_O_2_ (W/W) for 30 min and then germinated in a plastic basket (25 cm × 18 cm) with mesh. After the seedlings had developed an average of 2.5 visible leaves, they were transplanted to a 7-L plastic box containing a quarter-strength nutrient solution. Three days later, the seedlings were transferred to a one half-strength nutrient solution, and after five days, the seedlings were supplied with full–strength (2.8 mM·N) nutrient solution for one week. One week later, the seedlings were supplied with low-N, 0.7 mM and high-N, 7 mM. The N sources were equimolar amounts of (NH_4_)_2_SO_4_ and Ca(NO_3_)_2_. In addition, the macronutrients in the solution were listed as follows (mM): 0.32 P as KH_2_PO_4_, 1.02 K as K_2_SO_4_ and KH_2_PO_4_, and 1.65 Mg as MgSO_4_. The micronutrients were (μM) as follows: 35.8 Fe as Fe–EDTA, 9.10 Mn as MnCl_2_·4H_2_O, 0.52 Mo as (NH_4_)_6_Mo_7_O_24_·4H_2_O, 18.5 B as H_3_BO_3_, 0.15 Zn as ZnSO_4_·7H_2_O, 0.16 Cu as CuSO_4_·5H_2_O, and 100 Si as Na_2_SiO_3_·9H_2_O. CaCl_2_ was added to solutions to compensate for the lower Ca in the low-N-content solution. A nitrification inhibitor (Dicyandiamide, DCD) was added to each nutrient solution to prevent the oxidation of ammonium. The nutrient solutions were changed every three days, and the pH was adjusted to 5.50 ± 0.05 every day with HCl or NaOH.

The temperature in the glasshouse was maintained at 30 °C during the day and 18 °C at night. Light was supplied by SON-T AGRO 400 W bulbs; the light intensity was maintained at a minimum of 1000 µmol·photons·m^−2^·s^−1^ (photosynthetically active radiation) at the leaf level using a 14 h photoperiod.

### 4.2. Gas Exchange Measurement of Newly Expanded Leaves

After 30-day with high-N and low-N treatment, the light-saturated photosynthetic rate of newly expanded leaves was measured by Li-Cor 6400 portable photosynthesis system. Leaf temperature during the measurement was maintained at 28 °C and photosynthetic photon flux density (PPFD) was 1500 μmol·m^−2^·s^−1^. Meanwhile, stomatal conductance (g_s_) and transpiration rate were recorded.

### 4.3. Leaf Hydraulic Conductance (K_leaf_) Mesurement of Newly Expanded Leaves

After 30 days with high-N and low-N treatment, the evaporative method was used to determine newly expanded leaf hydraulic conductance (K_leaf_) based on Martre et al. [45]. K_leaf_ was calculated by equation: K_leaf_ = E/(Ψ_solution_ − Ψ_leaf_), where E was the leaf transpiration rate measured by Li-cor 6400, Ψ_solution_ was 0 for hydroponic culture, Ψ_leaf_ was the leaf water potential measured by WP4 Dewpoint Potential Meter (Decagon Devices, Houston, TX, USA). After gas exchange measurement, newly expanded leaf was cut into small sections and water potential was measured immediately. In this study, only SY63 and YD6 were measured based on previous studies [3,4,42]. Under high-N supply, Ψ_leaf_ decreased in SY63 [4], and aquaporin expression did not increase in high-N supply leaves [3]. High-N supply did not decrease Ψ_leaf_ in YD6 and high-N increased aquaporin expression in YD6 [4,46]. Thus it is interesting to compare K_leaf_ between SY63 and YD6.

### 4.4. Real-Time-Quantitative PCR (RT-qPCR)

At around 11:00 a.m., newly expanded leaf was collected and immediately put in liquid nitrogen, and stored at −80 °C until RNA extraction. The total RNA was extracted with TRIzol reagent (Invitrogen) according to the manufacturer’s instructions. cDNA was synthesised using PrimeScript™ RT reagent Kit with gDNA Eraser (Takara, Dalian, China). RT-qPCR was performed using the ABI 7500 Real-Time PCR system, with SYBR green master mix (SYBR^®^ Premix Ex Taq™ II (Tli RNaseH Plus); Takara, Dalian, China). The primers for RT-qPCR were according to Sakurai-Ishikawa et al. [23], and 18sRNA was used as the housekeeping gene. The relative gene expression was calculated with 2^−Δ*C*t^ method.

### 4.5. Electron Microscopy

Approximately 1~2 mm^2^ leaf sections were cut from the middle of newly expanded leaves, fixed with 2.5% glutaraldehyde (0.1 mol L^−1^ phosphate buffer, pH = 7.4), and evacuated by syringe to remove air inside leaves. The leaves were post-fixed with 2% osmium tetroxide, followed by dehydrating in a graded acetone series and embedding in Epon 812. Samples were cut on a Power Tome-XL ultra microtome, stained with 2% uranyl acetate, and examined with H-7650 transmission electron microscope (TEM, Hitachi-Science & Technology, Tokyo, Japan).

The intercellular air space (*f*_IAS_ (%) = (intercellular air space/mesophyll cells space) × 100) and cell wall thickness were calculated by using Image J (National Institutes of Health, http://rsb.info.nih.gov/ij) with images from TEM.

### 4.6. Statistical Analysis

Student’s *t* test was used to assess the differences between the treatments using the JMP 9 statistical software package (SAS Institute, Cary, NC, USA). The significant differences were indicated with *, ** and ***, in the levels of *p* < 0.05, *p* < 0.01 and *p* < 0.001, respectively.

## 5. Conclusions and Perspectives

In this study, high-N supply increased the photosynthetic rate and transpiration rate. Meanwhile, the expression of *PIPs* and *TIP* was higher in high-N than low-N plants in YD6, ZD11 and JY418 variants, but not in SY63. In comparing K_leaf_ values, these was shown to be increased by high-N supply in YD6, but decreased under high-N than low-N in the SY63 variant. Under high-N supply, the decrease of K_leaf_ was due to low leaf water potential, low aquaporin expression and high cell wall thickness in SY63. In conclusion, the expression change of aquaporins could affect K_leaf_ and transpiration. A feedback effect of transpiration would regulate aquaporin expression.

The present results imply coordination between gas exchange with leaf hydraulic conductance, which is regulated by N supply. There was a limited increase of the photosynthetic rate under high-N supply. Photosynthetic nitrogen use efficiency (PNUE) decreased under high-N supply [3,24,42]. Modification of leaf hydraulic conductance might be useful to break the limited increase of the photosynthetic rate under high-N supply. Additionally, nitrogen’s effects on aquaporin expression and the transpiration rate may be used to improve crop yield for plant breeding.

## Supplementary Materials

Supplementary materials can be found at www.mdpi.com/1422-0067/19/1/256/s1.
