# Supplementary material for: Aquaporin Expression and Water Transport Pathways inside Leaves Are Affected by Nitrogen Supply through Transpiration in Rice Plants
Source: Int J Mol Sci. 2018 Jan 16;19(1):256. doi: 10.3390/ijms19010256 (PMC5796202; doi:10.3390/ijms19010256)
Supplement: Supplementary file 1 [file ijms-19-00256-s001.pdf]

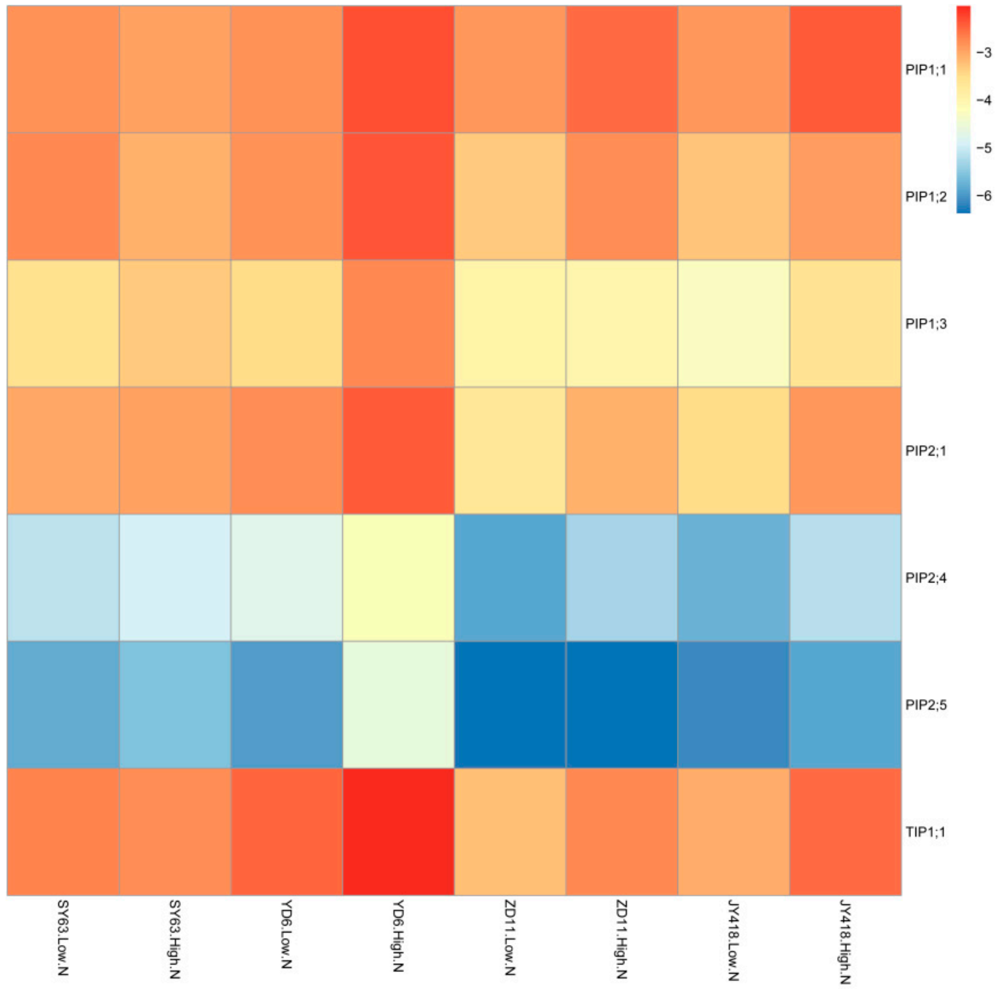

Figure S1. The expressions of six PIPs and one TIP in leaves supply with Low-N and High-N in four rice cultivars.

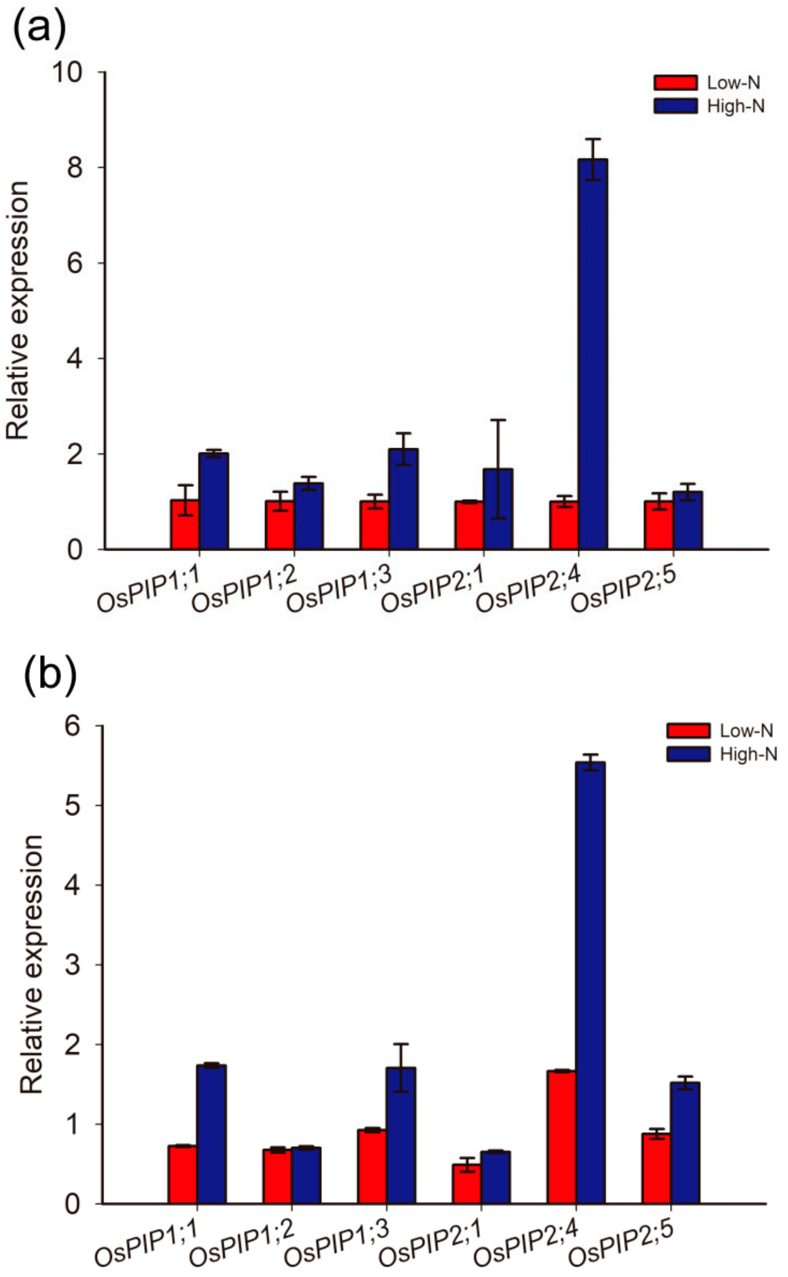

Figure S2. The expression of PIPs in roots supply with Low-N and High-N in SY63 and YD6.

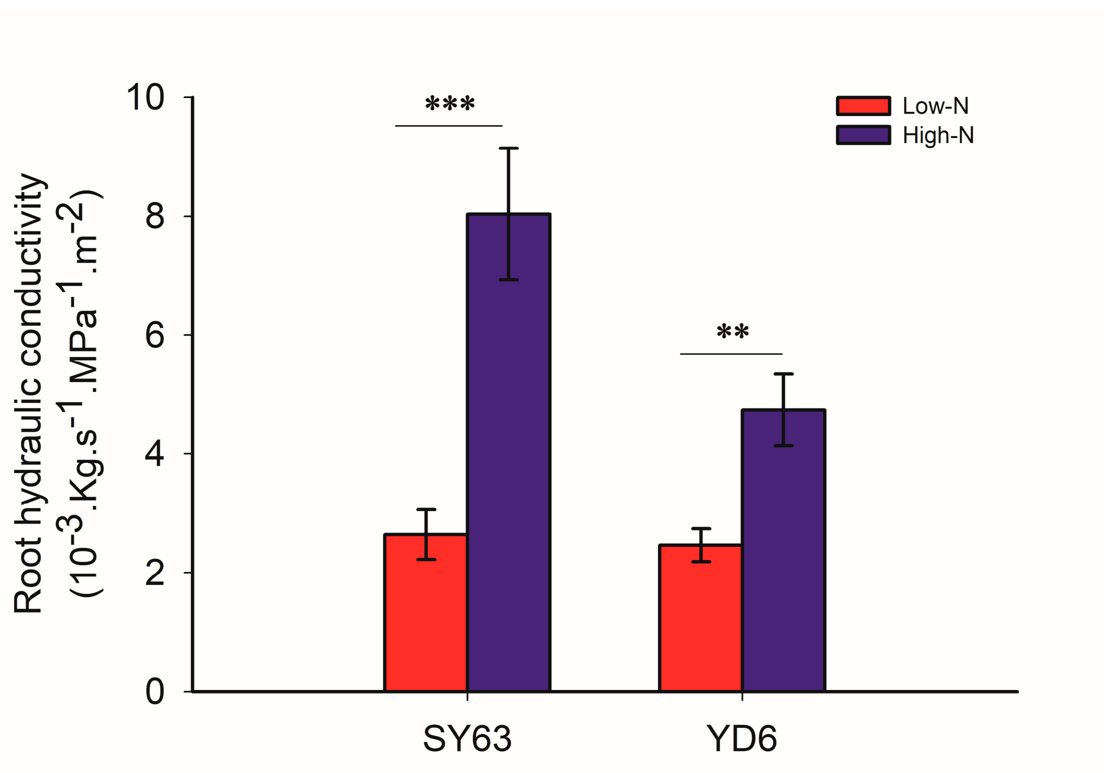

Figure S3. The effect of different nitrogen supply on root hydraulic conductivity (Lpr) in SY63 and YD6.
